# Interactive machine learning for soybean seed and seedling quality classification

**DOI:** 10.1038/s41598-020-68273-y

**Published:** 2020-07-09

**Authors:** André Dantas de Medeiros, Nayara Pereira Capobiango, José Maria da Silva, Laércio Junio da Silva, Clíssia Barboza da Silva, Denise Cunha Fernandes dos Santos Dias

**Affiliations:** 1 Agronomy Department, Federal University of Viçosa, Viçosa, Minas Gerais 36570-900 Brazil; 2 Center for Nuclear Energy in Agriculture (CENA), University of Sao Paulo (USP), Piracicaba, São Paulo 13416‐000 Brazil

**Keywords:** Plant physiology, Computational models, Machine learning

## Abstract

New computer vision solutions combined with artificial intelligence algorithms can help recognize patterns in biological images, reducing subjectivity and optimizing the analysis process. The aim of this study was to propose an approach based on interactive and traditional machine learning methods to classify soybean seeds and seedlings according to their appearance and physiological potential. In addition, we correlated the appearance of seeds to their physiological performance. Images of soybean seeds and seedlings were used to develop models using low-cost approaches and free-access software. The models developed showed high performance, with overall accuracy reaching 0.94 for seeds and seedling classification. The high precision of the models that were developed based on interactive and traditional machine learning demonstrated that the method can easily be used to classify soybean seeds according to their appearance, as well as to classify soybean seedling vigor quickly and non-subjectively. The appearance of soybean seeds is strongly correlated with their physiological performance.

## Introduction

Soybean [*Glycine max* (L.) Merrill] is one of the main commodities in world agribusiness. Soybean seeds are rich in amino acids, lipids, vitamins, and minerals and are an abundant source of proteins, constituting a key crop for global food security. Achieving high soybean yield depends on successful establishment of soybean plants, which requires use of high-quality seeds^1^.

Seed quality is very susceptible to environmental conditions and post-harvest procedures such as mechanical threshing and artificial drying^2^. These factors may lead to significant seed damage and to changes in seed appearance. Damage from mechanical kneading pressure, attack from pathogens, broken seeds, ruptured seed coats, moisture damage, and greenish seeds may comprise seed performance. Many efforts have been made to prevent this damage and improve seed quality. The identification of low-quality seed lots is particularly important. In most cases, this identification is based on visual inspection of seed lots and on chemical methods that are destructive, subjective, inconsistent, and time-consuming^3^.

Machine learning methods have supported a recent revolution in computer vision^4^. One recent and promising user-oriented machine learning approaches is Interactive Machine Learning (IML). IML approaches are defined by algorithms that can interact with both computational agents and human agents^5^. They aim to use human knowledge and skills in a timely and repeated manner to improve the accuracy of the models. IML approaches can therefore be effective in problems with small datasets or complex datasets when traditional machine learning methods become inefficient^6^. The combination of these machine learning algorithms with computer vision has brought new and promising perspectives for analyzing the quality of agricultural products, especially seeds^7–9^. With these technologies, many of the limitations now faced by traditional methods of visual seed inspection could be resolved.

Collaborative open-source software has offered powerful solutions in several research fields that use bioimages. These tools have become prominent due to their flexibility and transparency in dealing with new technologies^10^, and new possibilities of application are opening in as yet little explored areas. Among these tools, Ilastik is open-source software that allows the development of models based on interactive machine learning with images; it is easy to use and ideal for users without substantial computational knowledge^4^. This software has been used in recent studies to measure the confluence of Hep G2 cell culture in phase-contrast micrographs^11^, high-throughput screening for quantifying thrips damage^12^, quantification and spatial analysis of features in histological images of rodent brains^13^, and others. However, there is still no information on application of interactive machine learning via Ilastik in plant science and, in particular, in studies on seeds and seedlings.

Considering that the use of image analysis in seed science technology has led to considerable advances in seed quality assessment^14^, the Ilastik software could be integrated in this image analysis, which would make seed quality assessment even more efficient. Thus, the aim of this study was to propose an approach based on interactive and traditional machine learning methods to classify soybean seeds and seedlings according to their appearance and physiological potential. In addition, we correlated the appearance of seeds with their physiological performance.

## Materials and methods

### Plant material and image acquisition

Seven commercial soybean seed lots from production in the 2019/2020 crop season were acquired, from which 700 seeds were selected. The seeds were divided into seven groups according to physical, health, and physiological aspects. The groups were composed of mechanically damaged seeds, seeds infested by fungi, and green seeds (with low chlorophyll degradation). Initially, images of the seeds were obtained from an Epson Perfection V800 scanner. The seeds were tested for germination capacity by placing them on rolls of germination paper moistened with water in the amount of 2.5 times the dry weight of the paper and kept in a germinator (Mangelsdorf) at 25 °C for three days. The seedlings produced were individually evaluated through image analysis to determine seed vigor. Images of the seedlings were acquired through an HP Scanjet 200 scanner fastened in an inverted position within an aluminum box. Both seed and seeding images had 300 dpi resolution.

### Image pre-processing and segmentation

Image pre-processing and segmentation were performed with the Ilastik software^4^ using the pixel classification tool. The images were semantically segmented, and two segmentation classes were defined: “seed or seedling” and “background”. The features of the seeds or seedlings were based on color and pixel intensity and border and texture descriptors, computed as pre-smoothed filters with a sigma ranging from 0.3 to 1. To create the probability maps, the pixels belonging to the regions of each class were selected by painting brushstrokes of different colors directly on the input data. A Random Forest classifier was then used for pixel classification (Ilastik standard). The probability that the pixel belonged to the semantic segmentation classes (“seed/seedling” or “background”) was estimated for each pixel of the image. The trained classifier was applied to all images, individual seeds and seedlings, in batch mode, and the probability maps were exported for each image.

### Seed appearance and physiological quality classification

In this study, we developed independent classifications for soybean seed appearance and physiological quality, considering seedling growth and the non-germinated seed data. The methods used for both classification procedures were similar; therefore, the descriptions were presented only once.

### Seed appearance classes

Seeds were classified into seven different classes, based on visual inspection of each individual seed: (1) high-quality seed (HQS)—seed nearly round, firm, with smooth skin, and a single color; (2) mechanically kneaded seed (KNS)—seeds with irregular surfaces and surface depressions due to mechanical damage; (3) purple stained seed (PSS)—seeds with pink to light or dark purple discoloration, with size ranging from a small spot to covering the entire seed coat; (4) broken seed (BRS)—seeds with poor structural integrity; (5) seed coat tear (SCT)—seeds showing coat ruptures; (6) moisture damaged seed (MDS)—seeds showing wrinkles in the region opposite the hilum; (7) greenish seed (GRS)—seeds with a greenish seed coat or cotyledon.

### Physiological quality classes

The seedlings were placed in two classes: (1) vigorous seedlings (VSD)—morphologically healthy seedlings showing vigorous growth of the hypocotyl and roots; and (2) weak seedlings (WSD)—seedlings showing absence, underdevelopment, or deformation of some essential structure (cotyledons, primordial leaves, and roots). In addition, the class (3) non-germinated seeds (NGS)—seeds unable to germinate after three days under suitable conditions—was established.

### Interactive classification of seed appearance and vigor

Interactive classification was performed with the Ilastik software, using the inbuilt object classification tool. This step was the main human task in the application of interactive machine learning. First, the training images were input (10% of the total images were used) and their respective probability maps were obtained in the pre-processing stage. Then 103 variables^15^, all the features available in the Ilastik software, were calculated for each seed and seedling, including convex-hull, skeleton-based shape descriptors, and property intensity statistics. Finally, we trained the classifier by selecting known individual classes. This process was performed by clicking on the reference objects of the different classes. Immediately, the classification was made. That way, real-time classification allowed us to track the classification errors and to correct them. This approach differs from traditional machine learning, which requires a lot of effort to detect point flaws in classification and often requires significant amounts of training data^6^. It is important to highlight that, in this study, the human effort of performing the classifications was not evaluated, but it comprised only the initial steps, and once the classification was completed, the classifier could be used automatically.

Then, the trained classifier was applied to the entire set of images in batch mode. The descriptors and the individual prediction maps were exported in CSV and PNG file formats, respectively. This approach did not require hardware with a high level of processing capacity. We performed all classifications on an Intel CoreCPU i5‐42000 @ 1.60 GHz and 4 GB of RAM.

### External algorithms for seed appearance and physiological quality classification

The descriptors generated by Ilastik for each seed and seedling were used to develop classification models using traditional machine learning techniques based on three methods: Linear Discriminant Analysis (LDA), Random Forest (RF), and Support Vector Machine (SVM). The data were divided into training sets and validation sets, in the proportion of 70% and 30%, respectively. The three classifiers were developed with the R 3.6.3 software (R Core Team, 2019). For LDA, the Mass (https://cran.r-project.org/web/packages/MASS/index.html) package was used. For RF models, the cforest function of the partykit package (https://www.rdocumentation.org/packages/partykit) was used, with 500 decision trees and default hyperparameters. For SVM, the caret (https://cran.r-project.org/web/packages/caret/index.html) package was used, with a radial kernel and default parameters.

### Model validation

The models developed with the interactive method using the Ilastik software were validated using 90% of the data set. These data had not been used for training. The external models developed were validated through cross-validation (10-folds) and through an independent validation set that had not been used before. They were evaluated based on True Positive (TP), False Positive (FP), True Negative (TN), and False Negative (FN) data. The Accuracy, Kappa, Precision, Sensitivity, and Specificity metrics were calculated:

1 $$Accuracy=\frac{TP+TN}{TP+TN+FP+FN}$$

2 $$Kappa=2*\frac{TP*TN-FP*FN}{TP*FN+TP*FP+2*TP*TN+{FN}^{2}+FN*TN+{FP}^{2}+FP*TN}$$

3 $$Precision=\frac{TP}{TP+FP}$$

4 $$Sensitivity=\frac{TP}{TP+FN}$$

5 $$Specificity=\frac{TN}{TN+FP}$$

### Association of seed appearance and physiological quality

In order to understand the relationship between seed appearance and seed physiological quality, we made an association between seed classes and their respective seedling classes. Seedling length was measured and the data were used to obtain the growth, uniformity, and vigor indices and root length using the Vigor-S software^16^. Multivariate principal component analysis was applied to these data, adopting the seed classes as individuals and the seedling vigor indices as vectors.

## Results

### Machine learning models for seed classification

We developed and compared four different models to classify soybean seeds based on relevant visible aspects using a high-performance tool. The model based on interactive machine learning showed an overall accuracy of 0.83 (Table 1). The BRS and SCT classes had the highest hit rates, with sensitivity greater than 0.97. In contrast, the HQS and MDS classes had lower individual accuracy, with a higher rate of false-positives and false-negatives. At least 21% of HQS class seeds were confused with MDS class seeds, while the confusion increased to 30% in the opposite direction.

**Table 1:** Confusion matrices and metrics of the interactive classification of soybean seeds according to their visual appearance.

Class^a^	High-quality seed	Kneaded seed	Purple stained seed	Broken seed	Seed coat tear	Moisture damaged seed	Green-ish seed
	n = 630						
High-quality seed	70	1	3	0	2	27	0
Kneaded seed	0	76	17	0	0	0	6
Purple stained seed	1	2	64	0	0	0	2
Broken seed	0	0	0	87	0	1	0
Seed coat tear	0	3	3	3	88	1	2
Moisture damaged seed	19	0	1	0	0	61	3
Greenish seed	0	8	2	0	0	0	77
Accuracy	0.92	0.95	0.94	0.99	0.98	0.92	0.96
Kappa	0.68	0.78	0.77	0.97	0.91	0.65	0.85
Precision	0.68	0.77	0.93	0.99	0.88	0.73	0.89
Sensitivity	0.78	0.84	0.71	0.97	0.98	0.68	0.86
Specificity	0.94	0.96	0.99	1	0.98	0.96	0.98

The Random Forest classifier was applied and 10% of total images were used for training.

^a^In the columns are the true seed classes, and in the rows are the estimated classes.

The seed descriptors generated by the Ilastik software were tested applying three different machine learning methods (Table 2). In general, high performance was observed for all three models developed. The LDA method stood out, achieving an accuracy of 0.93 in the cross-validation set and 0.94 in the independent validation set, and a Kappa coefficient above 0.91. In independent validation, the classes SCT and BRS had the highest hit rates in the LDA, RF, and SVM models.

**Table 2:** The number of seeds correctly classified in each class and metrics for the external seed classification models using the seed descriptors generated by Ilastik software.

Method	Class	Training set	Cross-validation	Validation set
		(n = 490)		(n = 210)
		Hits (total)		
LDA	High-quality seed	68 (70)	–	29 (30)
	Kneaded seed	64 (70)	–	29 (30)
	Purple stained seed	69 (70)	–	28 (30)
	Broken seed	68 (70)	–	30 (30)
	Seed coat tear	70 (70)	–	30 (30)
	Moisture damaged seed	66 (70)	–	25 (30)
	Greenish seed	69 (70)	–	27 (30)
	Overall accuracy	0.98	0.93 ± 0.03	0.94
	Kappa	0.96	0.92 ± 0.04	0.93
	Precision	0.97	0.93 ± 0.04	0.94
	Sensitivity	0.97	0.93 ± 0.03	0.94
	Specificity	0.99	0.99 ± 0.01	0.99
RF	High-quality seed	64 (70)	–	25 (30)
	Kneaded seed	65 (70)	–	27 (30)
	Purple stained seed	70 (70)	–	28 (30)
	Broken seed	69 (70)	–	30 (30)
	Seed coat tear	70 (70)	–	29 (30)
	Moisture damaged seed	64 (70)	–	24 (30)
	Greenish seed	66 (70)	–	26 (30)
	Overall accuracy	0.96	0.89 ± 0.06	0.90
	Kappa	0.95	0.87 ± 0.07	0.88
	Precision	0.96	0.90 ± 0.06	0.90
	Sensitivity	0.96	0.89 ± 0.07	0.90
	Specificity	0.99	0.98 ± 0.07	0.98
SVM	High-quality seed	67 (70)	–	27 (30)
	Kneaded seed	63 (70)	–	26 (30)
	Purple stained seed	68 (70)	–	28 (30)
	Broken seed	68 (70)	–	30 (30)
	Seed coat tear	70 (70)	–	30 (30)
	Moisture damaged seed	64 (70)	–	24 (30)
	Greenish seed	67 (70)	–	26 (30)
	Overall accuracy	0.95	0.92 ± 0.03	0.91
	Kappa	0.95	0.91 ± 0.04	0.89
	Precision	0.95	0.92 ± 0.03	0.91
	Sensitivity	0.95	0.92 ± 0.03	0.90
	Specificity	0.99	0.99 ± 0.01	0.98

### Machine learning models for physiological quality classification

The physiological data were used for seed classification into three classes according to seed germination capacity and seedling growth: vigorous seedlings (VSD), weak seedlings (WSD), and non-germinated seeds (NGS). Four models were developed to that end—the first based on interactive machine learning and the others using the traditional machine learning approach to analyze the data generated by the Ilastik software. Note that although the data set in this study had 700 seeds, only 600 were used for development and evaluation of these models. The BRS class was excluded since it is not usually used to assess seed vigor.

A high precision classification model within the interactive Ilastik machine learning method was used (Table 3). The model achieved average accuracy of 0.97, and values above 0.92 for kappa, precision, sensitivity, and specificity metrics for the VSD class. The highest rate of false-negatives was found in the WSD class and the lowest rate in the VSD class.

**Table 3:** Confusion matrices and metrics of the interactive Ilastik machine learning classification of soybean seeds according to their physiological quality.

Class^a^	Vigorous seedling	Weak seedling	Non-germinated seed
	n = 600		
Vigorous seedling	230	18	0
Weak seedling	1	157	9
Non-germinated seed	0	10	174
Accuracy	0.97	0.94	0.97
Kappa	0.93	0.85	0.93
Precision	0.93	0.94	0.95
Sensitivity	0.99	0.85	0.95
Specificity	0.95	0.98	0.98

^a^In the columns are the true seed classes, and in the rows are the estimated classes.

Models based on external classification were also satisfactory (Table 4). For those models, the data generated by the Ilastik software for seedlings and non-germinated seeds were used. The training set comprised 70% of the data. The Random Forest method performed slightly better than the others in the cross-validation and independent validation sets, showing 0.93 and 0.94 accuracy, respectively. The hit rate for each class varied according to the classifier, but in general, there was a more significant classification error for the WSD class.

**Table 4:** The number of seedlings classified correctly in each class and metrics for external classification using Ilastik descriptors of soybean seeds and seedlings according to their physiological quality.

Method	Class	Training set	Cross-validation	Validation set
		(n = 422)		(n = 178)
		Hits (Total)		
LDA	Vigorous seedlings	159 (162)	–	64 (69)
	Weak seedlings	122 (131)	–	44 (55)
	Non-germinated seeds	129 (129)	–	52 (54)
	Overall accuracy	0.97	0.92 ± 0.04	0.90
	Kappa	0.96	0.89 ± 0.06	0.85
	Precision	0.97	0.92 ± 0.04	0.90
	Sensitivity	0.97	0.93 ± 0.04	0.90
	Specificity	0.99	0.96 ± 0.02	0.95
RF	Vigorous seedlings	160 (162)	–	67 (69)
	Weak seedlings	120 (131)	–	49 (55)
	Non-germinated seeds	128 (129)	–	52 (54)
	Overall accuracy	0.97	0.93 ± 0.03	0.94
	Kappa	0.95	0.89 ± 0.04	0.92
	Precision	0.97	0.93 ± 0.03	0.94
	Sensitivity	0.97	0.93 ± 0.03	0.94
	Specificity	0.98	0.96 ± 0.02	0.97
SVM	Vigorous seedlings	158 (162)	–	60 (69)
	Weak seedlings	110 (131)	–	42 (55)
	Non-germinated seeds	127 (129)	–	48 (54)
	Overall accuracy	0.94	0.89 ± 0.03	0.84
	Kappa	0.90	0.83 ± 0.04	0.76
	Precision	0.94	0.89 ± 0.02	0.84
	Sensitivity	0.93	0.89 ± 0.03	0.84
	Specificity	0.97	0.94 ± 0.02	0.92

### Classification summary

**Figure 1:**
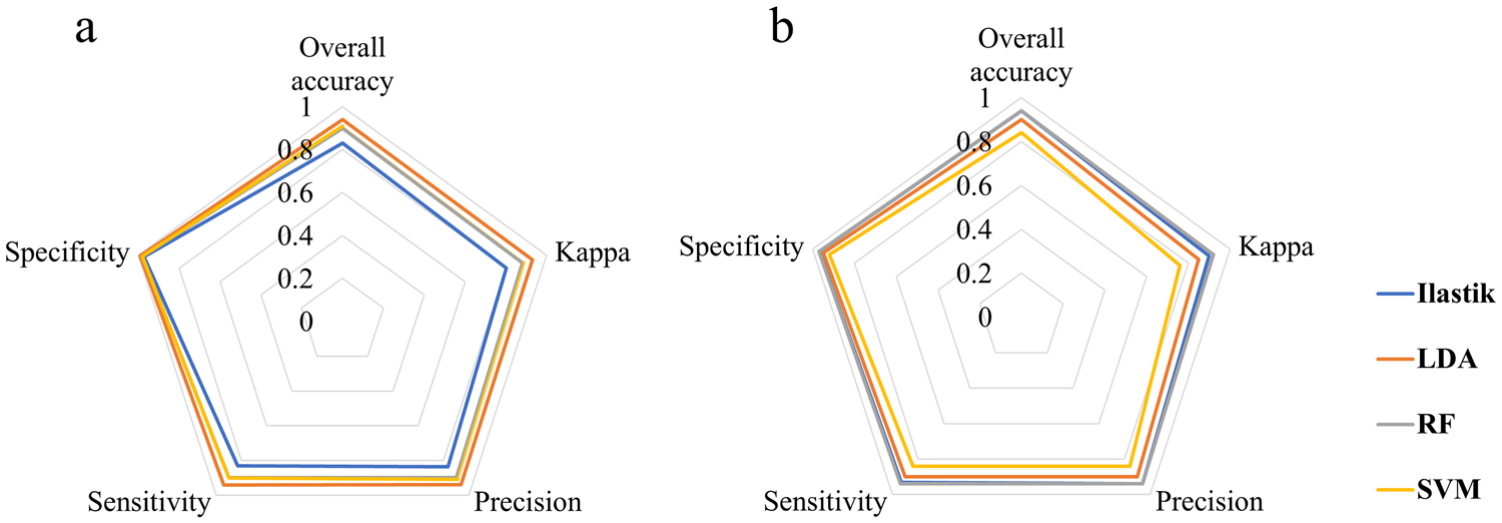
Star plot for the metrics of the machine learning classifiers tested. Classifications based on seed appearance (**a**) and physiological quality (**b**).

In general, the classifications based on interactivity machine learning and the traditional approach were accurate. However, especially for classifications based on seed appearance, the external classification models performed better (Fig. 1a). For those models, it is important to emphasize that 70% of the data was used for training; thus, we expected higher accuracy. For classification based on seed physiological quality, the models showed similar performance (Fig. 1b). Note that the Ilastik method was slightly better (higher values) in most metrics than the LDA and SVM algorithm, even using only 10% of the data for training, versus 70% in the external classification method.

### Relationship between seed appearance and seedling growth

**Figure 2:**
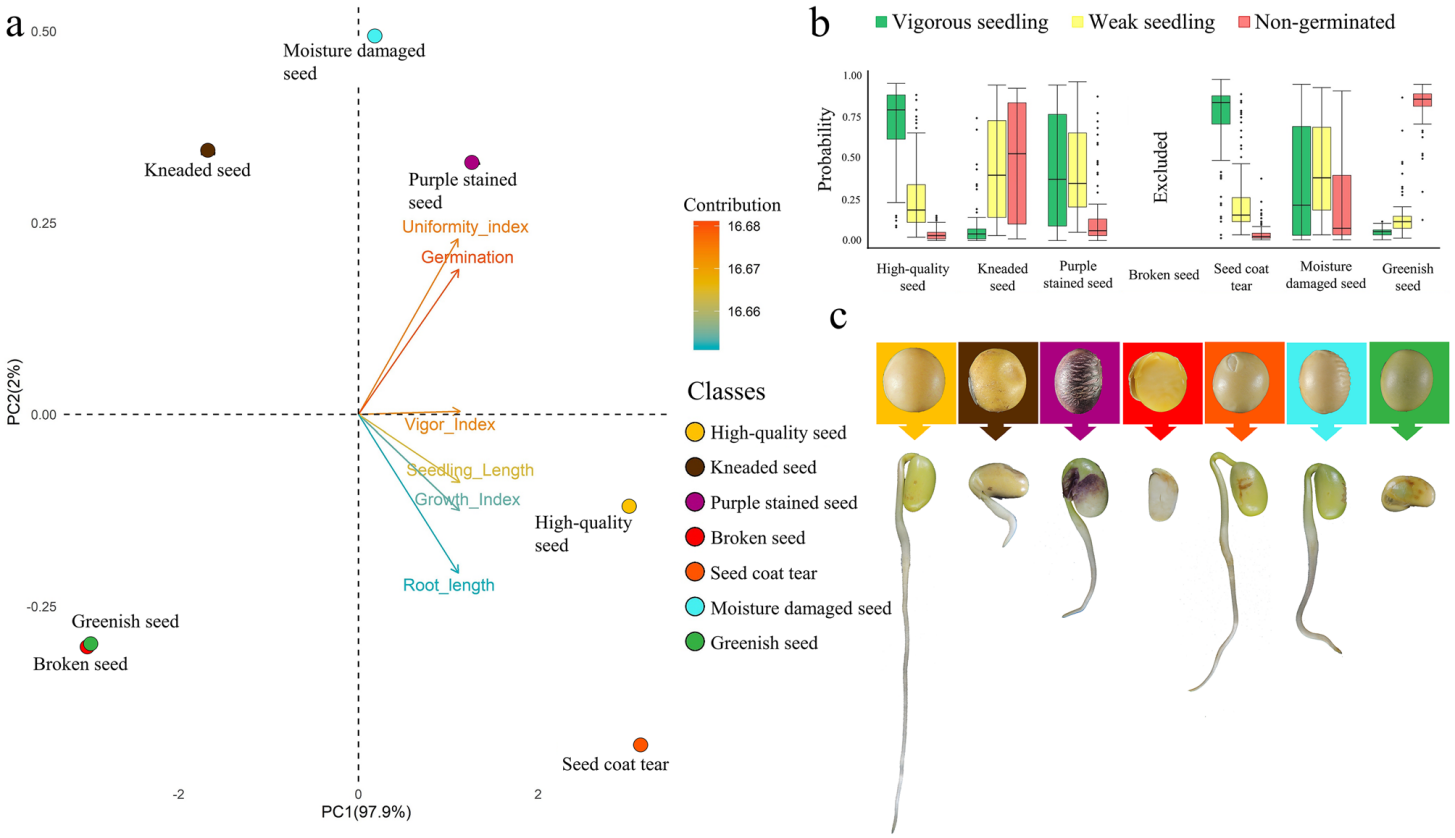
Relationship between seed appearance and seedling growth. Biplot of principal component analysis showing the importance of the seed quality parameters for class dispersion (**a**), probability of generating vigorous seedlings and weak seedlings or lack of germination of individual seeds according to their class (**b**), and seed appearance and the predominant aspect of the seedlings in each seed class (**c**).

The seeds exhibited different physiological performance according to their appearance (Fig. 2). In multivariate principal component analysis, the physiological quality vectors were positioned to the right of the ordering diagram, indicating that individuals located in negative scores of PC1 had significantly lower values for these variables (Fig. 2a). In more detail, HQS and SCT classes showed higher values of seedling length and higher vigor, uniformity, and growth indices (Fig. 2a). For those classes, all seeds germinated and generated a high proportion of vigorous seedlings (Fig. 2b). In addition, in the PSS and MDS classes, the seeds generated mainly vigorous seedlings and weak seedlings. The MDS also had a high proportion of non-germinated seeds. For the KNS class, the seeds generated mostly weak seedlings or did not germinate. Finally, the seeds of the GRS class mostly did not germinate. The seedling appearance that predominated in each seed class is shown in Fig. 2c.

## Discussion

The quality of soybean seed has a direct impact on its market price and affects seedling establishment in the field^7^. Visual-based inspection methods are currently used by laboratories to precisely evaluate soybean seed quality, including seedling evaluations. However, these methods are subjective, inconsistent, time-consuming, and usually destructive^3^. In this study, we present an approach based on interactive and traditional machine learning methods to classify soybean seeds and seedlings automatically, efficiently, and without costly resources.

The proposed methods were highly satisfactory. The accuracies found in discriminating seeds in their different appearance classes were high, ranging from 0.92 to 0.99 for the interactive machine learning model and greater than 0.90 of overall accuracy in the independent validation set for external classification (Tables 1, 2). These results indicate high potential for practical application of these methods. In this study, we used several seed lots, which is a good first step toward generalization, and it raises hope concerning possible generalization for industrial purposes. The models developed can easily be inserted in the quality control programs of seed companies or seed analysis labs, or even in studies in research centers. We are aware that this approach does not entirely dispense with human effort, as some manual tests are required to meet ISTA standards^17^, but it can be used in the process of sorting lots regarding seed quality and identifying reasons for loss of quality rapidly and accurately. It is noteworthy that this is a pioneering study using interactive machine learning methods for classifying soybean seeds according to their appearance.

In previous studies, researchers have tried to identify damage to soybean seeds using different techniques. Lin et al. described the representation of jointly multi-modal bag-of-feature (JMBoF) to inspect the appearance quality of post-harvest dry soybean seeds using images obtained in the visible spectrum. The proposed algorithm reached an accuracy of 82% in the test set. Mahajan et al. used RGB and X-ray images to assess the physical purity, viability, and vigor of soybean seeds. The classifier developed that was based on a neural network achieved an accuracy of 91%. Momin et al. developed a machine vision system to detect impurities in samples of harvested soybeans and achieved accuracy ranging from 75 to 98%. Liu et al. developed a system to identify and eliminate damaged soybean seeds using computer vision, image-processing technologies, neural networks, and automated mechanical control. The system showed an average recognition accuracy of 97%. Several other techniques used to assess soybean seed quality are Raman spectroscopy^18,19^, near-infrared spectroscopy^20,21^, and nuclear magnetic resonance^22,23^.

The machine learning methods applied in this study were efficient for classification of seed physiological quality. We found overall accuracy of 0.94 in the interactive method and accuracy of up to 0.94 in the external classification using the independent validation set for RF (Tables 3, 4). This technique opens new perspectives for rapid analyses of soybean seed vigor and enables soybean seedling phenotyping for genetic studies. Other software programs have been developed to analyze soybean seed quality, mainly considering the length of seedlings, such as SVIS^24^, VIGOR-S^16^, SAPL^25^, and GroundEye (https://www.tbit.com.br/). However, some software programs are commercial products, which restricts their use. Here, we present a free and efficient alternative for seed/seedling analysis that can be used by many researchers, laboratories, and institutions interested in application of a highly efficient method for vigor classification of soybean seeds and seedlings.

**Figure 3:**
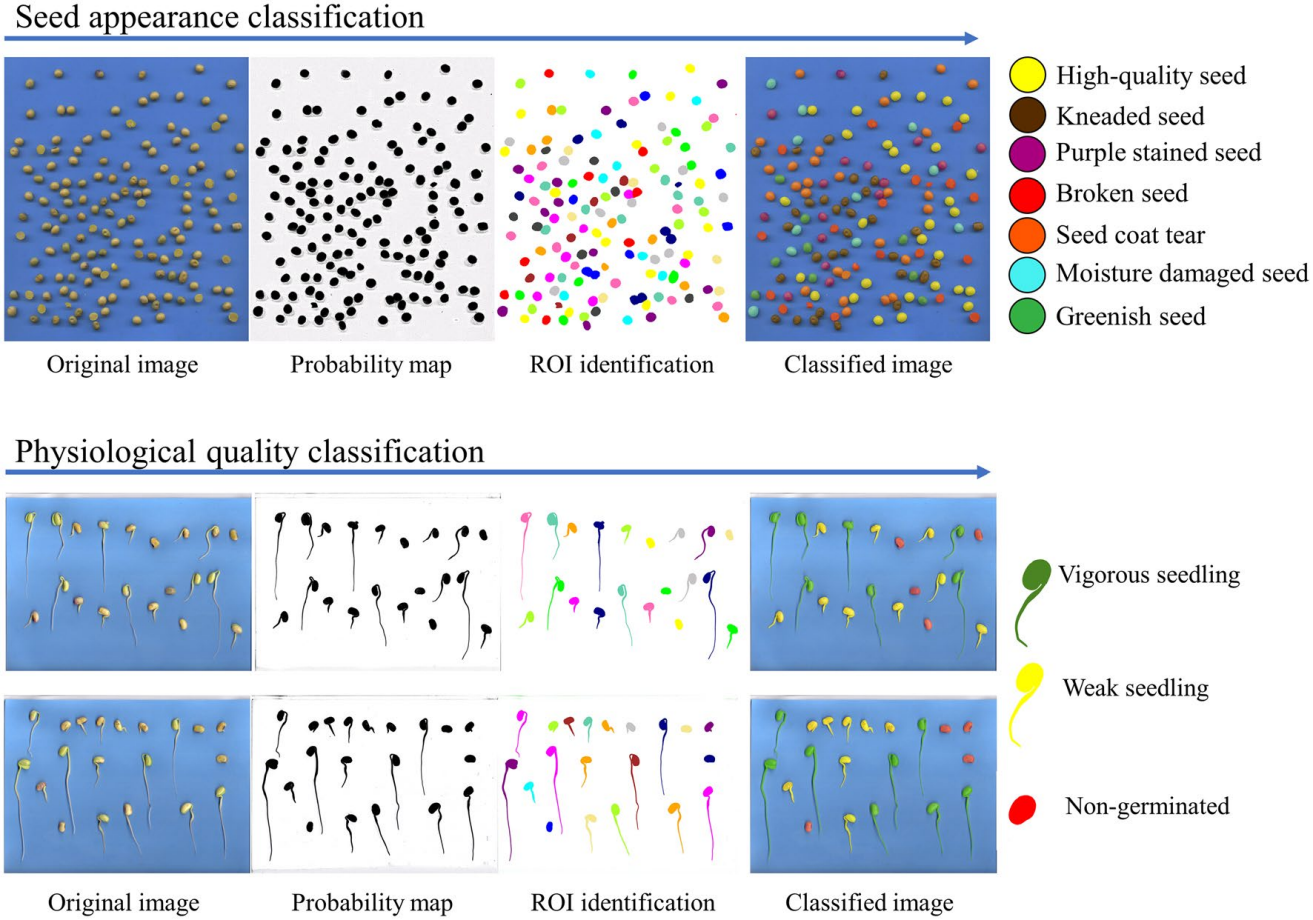
Representation of interactive machine learning steps and physiological quality classification in soybean seeds.

A summary of the steps of the interactive method applied to soybean seed and seedling classification is shown in Fig. 3. Image acquisition is made on a blue background to facilitate segmentation and to improve the ROI probability map. The identification of individual seeds and seedlings can be seen immediately after the probability map. Prediction of the classification of each seed or seedling is shown by the color of the respective groups.

The models developed can be improved to deal with more classes, and the Ilastik software provides all the tools necessary for this. The entire workflow of Ilastik (generic segmentation resources, nonlinear classifiers, probabilistic graphical models) is wrapped in an intuitive interface for interactive classifier training and post-processing of segmentation and classification algorithms^4^.

The machine learning algorithms applied to the external classification in this study have been used for complex data sets of plant phenotyping^26^. Methods such as LDA, RF, and SVM have been successfully used in plant science. The LDA is a popular machine learning algorithm that increases the distance between classes and reduces separability within the class, linearly combining features and creating limit estimates. The germination and vigor of *Jatropha curcas* seeds were accurately predicted using the LDA classifier combined with morphometric and tissue integrity resources obtained from X-ray images of seeds^14^. The RF, for its part, is a non-linear classifier formed by many decision trees. It generates the final classification based on a voting system for each tree, and in the end, the algorithm selects the most voted class^27,28^. In maize production areas in China, an RF-based classifier was developed using high-resolution remote-sensing images. The classifier was able to differentiate fields of seed and grain production and identify maize varieties^29^. By 4, the interactive machine learning method of Ilastik uses an RF classifier with 100 trees for pixel classification^4^. Lastly, SVM is a supervised algorithm that projects data into a higher dimensional resource space and identifies a hyperplane to separate classes with the most significant possible margin. The benefit of a hyperplane is its robustness to extreme values, considerably reducing false classifications^30^. In addition, in a recent study on individual plants in *Arabidopsis thaliana* under different levels of various abiotic stresses, an SVM algorithm using microRNA concentrations as input resources was able to predict plant stress with high precision (R^2^ = 0.96)^31^. These studies show the great potential of machine learning techniques.

Although our results were very promising, some limitations were noticed. The combined presence of different classes in the same individual seed is common in soybean. For example, a seed can be greenish and mechanically kneaded. This may have negative implications for classification if the objective is to accurately quantify individual damages. Another limitation is related to the acquisition method. Although the use of 2D scanners and cameras are the most accessible and straightforward approach to obtain images, the 2D image does not cover all the seed faces. In our study, this limitation caused confusion between the HQS and MDS classes (Table 1) since the moisture damage in some cases was not very evident. In future studies, 3D images could be applied.

Genetic and environmental factors regulate seed appearance, which is strongly correlated with seed physiological performance, as shown in the present study (Fig. 2). Interestingly, seeds with ruptured seed coats showed high vigor, which was similar to the high-quality seeds. It is believed that these ruptures are caused by genetic factors associated with environmental conditions during seed maturation. Although it is very common, there are few reports on the effect of this trait on seed physiological quality^32^.

Furthermore, the mechanically kneaded seeds resulted in a high number of non-germinated seeds and weak seedlings (Fig. 2b). This damage is caused by mechanical impacts during seed harvesting or processing. Depending on the location of the damage, the seeds may generate an abnormal seedling or not germinate at all. Seeds with extremely low moisture content are more susceptible to breakage during mechanical operations at harvest and in processing. These seeds were in the BRS class, which had practically no germinated seeds.

Purple stained seeds generally resulted in weak seedlings (Fig. 2b). This characteristic is mainly caused by the fungi *Cercospora kikuchii*. Previous reports showed a weak correlation between this characteristic and seed physiological quality, though it does compromise seed marketing.

Environmental conditions during seed production and mainly after seed maturation can lead to moisture damage. These seeds generally have low seed quality^33^, which leads to weak seedlings and a significant number of non-germinated seeds, as observed in the present study (Fig. 2b). Furthermore, high temperatures and water deficit during seed maturation can lead to green seed formation. These seeds have a low rate of seed germination (Fig. 2b).

Fast assessment of seed quality is essential for the seed industry. Rapid decision making regarding disposal or destination of seed lots saves time and resources. Thus, tools that can accurately evaluate seed lots and identify seeds of low physiological quality is of great importance. This study showed that it is possible to classify seeds according to their appearance, and these characteristics are strongly correlated with their physiological potential. Therefore, the proposed approach has potential for application in soybean seed lot classification for non-destructive, non-subjective, and fast screening of seeds. In addition, this method was able to classify soybean seedling vigor effectively and precisely.

## Conclusions

The interactive machine learning method for classification of soybean seeds from their appearance is highly accurate. This approach effectively identifies seeds with damage and classifies seedlings in vigor levels. The use of LDA, RF, and SVM algorithms is recommended for classifying soybean seeds and seedlings based on data generated with the Ilastik software. Soybean seeds with changes in chlorophyll degradation, fungal stains, and mechanical damage have low physiological quality.

## Supplementary information

Supplementary Information

## References

[1] Finch-Savage WE, Bassel GW (2016). Seed vigour and crop establishment: Extending performance beyond adaptation. J. Exp. Bot..

[2] Esteve Agelet L, Gowen AA, Hurburgh CR, O’Donell CP (2012). Feasibility of conventional and roundup ready soybeans discrimination by different near infrared reflectance technologies. Food Chem..

[3] Liu D (2015). Discriminating and elimination of damaged soybean seeds based on image characteristics. J. Stored Prod. Res..

[4] Berg S (2019). Ilastik: Interactive machine learning for (bio)image analysis. Nat. Methods.

[5] Holzinger A (2016). Interactive machine learning for health informatics: When do we need the human-in-the-loop?. Brain Inform..

[6] Holzinger A (2019). Interactive machine learning: Experimental evidence for the human in the algorithmic loop. Appl. Intell..

[7] Lin P (2019). Rapidly and exactly determining postharvest dry soybean seed quality based on machine vision technology. Sci. Rep..

[8] Mahajan S, Mittal SK, Das A (2018). Machine vision based alternative testing approach for physical purity, viability and vigour testing of soybean seeds (*Glycine max*). J. Food Sci. Technol..

[9] Momin MA, Yamamoto K, Miyamoto M, Kondo N, Grift T (2017). Machine vision based soybean quality evaluation. Comput. Electron. Agric..

[10] Dietz C (2020). Integration of the ImageJ ecosystem in the KNIME analytics platform. Front. Comput. Sci..

[11] Yordanov YI (2020). Hep G2 cell culture confluence measurement in phase-contrast micrographs—A user-friendly, open-source software-based approach. Toxicol. Mech. Methods.

[12] Visschers IGS, van Dam NM, Peters JL (2018). An objective high-throughput screening method for thrips damage quantitation using Ilastik and ImageJ. Entomol. Exp. Appl..

[13] Yates SC (2019). QUINT: Workflow for quantification and spatial analysis of features in histological images from rodent brain. Front. Neuroinform..

[14] de Medeiros AD, Pinheiro DT, Xavier WA, da Silva LJ, dos Dias DCFS (2020). Quality classification of *Jatropha curcas* seeds using radiographic images and machine learning. Ind. Crops Prod..

[15] Ilastik. Ilastik workflows. *lastik.org.* https://www.ilastik.org/documentation/objects/objects#from-segmentation-to-objects---object-feature-selection-applet (2020). Accessed 14 Jan 2020.

[16] Castan DOC, Gomes-Junior FG, Marcos-Filho J (2018). Vigor-S, a new system for evaluating the physiological potential of maize seeds. Sci. Agric..

[17] Boelt B (2018). Multispectral imaging—A new tool in seed quality assessment?. Seed Sci. Res..

[18] Lee H (2013). Prediction of crude protein and oil content of soybeans using Raman spectroscopy. Sens. Actuators B Chem..

[19] Schulmerich MV (2012). Protein and oil composition predictions of single soybeans by transmission Raman spectroscopy. J. Agric. Food Chem..

[20] Al-Amery M (2018). Near-infrared spectroscopy used to predict soybean seed germination and vigour. Seed Sci. Res..

[21] Kusumaningrum D (2018). Non-destructive technique for determining the viability of soybean (*Glycine max*) seeds using FT-NIR spectroscopy. J. Sci. Food Agric..

[22] Pietrzak LN, Frégeau-Reid J, Chatson B, Blackwell B (2002). Observations on water distribution in soybean seed during hydration processes using nuclear magnetic resonance imaging. Can. J. Plant Sci..

[23] Krishnan P, Joshi DK, Maheswari M, Nagarajan S, Moharir AV (2004). Characterisation of soybean and wheat seeds by nuclear magnetic resonance spectroscopy. Biol. Plant..

[24] Sako Y, Mcdonald MB, Fujimura K, Evans AF, Bennett MA (2001). A system for automated seed vigour assessment. Seed Sci. Technol..

[25] de Medeiros AD, Pereira MD (2018). SAPL : a free software for determining the physiological potential in soybean seeds. Pesqui. Agropecuária Trop..

[26] Rahaman MM, Chen D, Gillani Z, Klukas C, Chen M (2015). Advanced phenotyping and phenotype data analysis for the study of plant growth and development. Front. Plant Sci..

[27] Geurts P, Irrthum A, Wehenkel L (2009). Supervised learning with decision tree-based methods in computational and systems biology. Mol. Biosyst..

[28] Rahaman MM, Ahsan MA, Chen M (2019). Data-mining techniques for image-based plant phenotypic traits identification and classification. Sci. Rep..

[29] Zhang L (2020). Identification of seed maize fields with high spatial resolution and multiple spectral remote sensing using random forest classifier. Remote Sens..

[30] Lu Q (2020). Support vector machine approach for model-plant mismatch detection. Comput. Chem. Eng..

[31] Vakilian KA (2020). Machine learning improves our knowledge about miRNA functions towards plant abiotic stresses. Sci. Rep..

[32] Machado BR (2019). Effect of tear/crack on soybean (*Glycine max*) seed coat, physiological quality and pathology of the seed. Aust. J. Crop Sci..

[33] Ebone LA, Caverzan A, Chavarria G (2019). Physiologic alterations in orthodox seeds due to deterioration processes. Plant Physiol. Biochem..

