# Supplementary material for: Interactive machine learning for soybean seed and seedling quality classification
Source: Sci Rep. 2020 Jul 9;10:11267. doi: 10.1038/s41598-020-68273-y (PMC7347887; doi:10.1038/s41598-020-68273-y)
Supplement: Supplementary file 1 — Supplementary Information [file 41598_2020_68273_MOESM1_ESM.docx]

**Interactive machine learning for soybean seed and seedling quality classification**

André Dantas de Medeiros^1^*, Nayara Pereira Capobiango^1^, José Maria da Silva^1^, Laércio Junio da Silva^1^, Clíssia Barboza da Silva^2^ and Denise Cunha Fernandes dos Santos Dias^1^

^1^Agronomy Department, Federal University of Viçosa, 36570-900, Viçosa, Minas Gerais, Brazil.

^2^Center for Nuclear Energy in Agriculture (CENA), University of Sao Paulo (USP), 13416‐000, Piracicaba, São Paulo, Brazil

**Supplementary Figure**

**
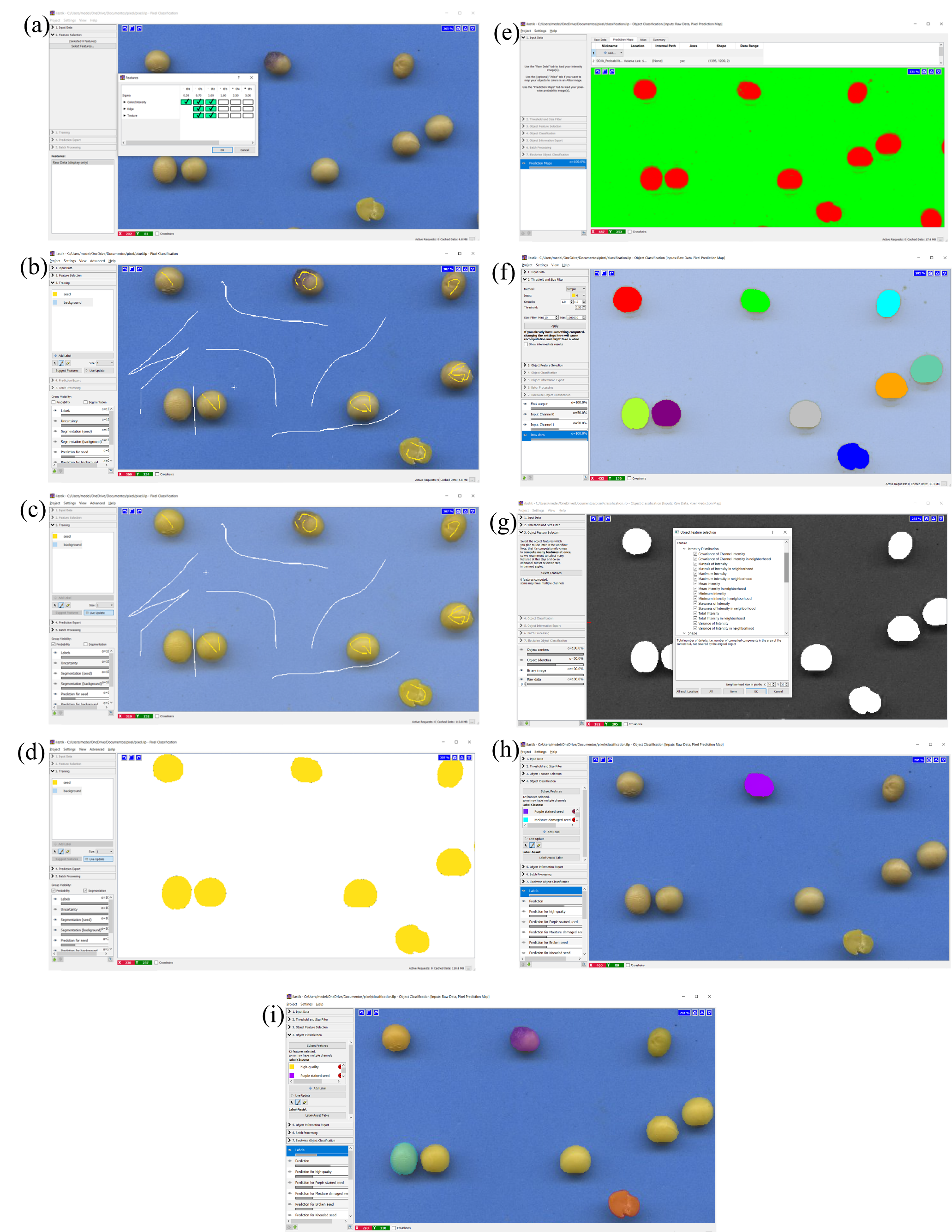
**

**Supplementary Figure 1.** Illustrative details of pixel classification sequence and object classification. (a) **Feature Selection, (b) p**ainting brush strokes, (c) probability map visualization, (d) segmentation view, (e) import of images and probability maps, (f) ROI identification, (g) f**eature selection, (h) k**nown individual classes selection, (i) probability maps of the object classification.
